# Efficacy of a virtual multimodal exercise program on postural awareness and digital eye strain in university students: a pilot study

**DOI:** 10.1590/1806-9282.20251229

**Published:** 2026-06-19

**Authors:** Ayça Aracı, Havva Nur Mutlu, Selenay Akgün, Ali Kurt

**Affiliations:** 1 Alanya Alaaddin Keykubat University, Faculty of Health Science, Department of Physiotherapy and Rehabilitation – Alanya, Türkiye.; 2 Alanya Alaaddin Keykubat University, Faculty of Medicine, Department of Ophthalmology – Alanya, Türkiye.

**Keywords:** Exercise therapy, Posture, Asthenopia, Anxiety, Students

## Abstract

**OBJECTIVE:**

Excessive screen exposure is associated with musculoskeletal discomfort, postural alterations, visual fatigue, and increased anxiety among young adults. This study investigated the effects of a virtually delivered multimodal exercise program integrating sensorimotor, oculomotor, and postural components on postural awareness, musculoskeletal complaints, visual fatigue, and anxiety among screen-exposed university students and staff.

**METHODS:**

Fifty-seven participants (mean age: 23.35±1.2 years) with ≥6 h of daily screen use completed an 8-week program consisting of twice-weekly live virtual sessions and daily video-guided exercises. Outcomes included the Postural Habits and Awareness Scale, Cornell Musculoskeletal Discomfort Questionnaire, Rapid Office Strain Assessment, ASTHENOPIA Eye Fatigue Scale, and State-Trait Anxiety Inventory. Pre–post differences were analyzed using paired-sample t-tests and Wilcoxon signed-rank tests.

**RESULTS:**

Significant improvements were observed in Postural Habits and Awareness Scale Factor 1 and Factor 3 (p<0.05), accompanied by reduced musculoskeletal discomfort across all Cornell Musculoskeletal Discomfort Questionnaire sections (p<0.05). Visual fatigue decreased substantially (z=-4.185; p<0.001), whereas Rapid Office Strain Assessment scores showed no significant change (p>0.05). State anxiety increased slightly (p<0.05), while trait anxiety remained stable.

**CONCLUSION:**

This preliminary single-group study suggests that virtual multimodal exercise programs may be effective, accessible preventive strategies for reducing musculoskeletal symptoms, enhancing posture awareness, and alleviating visual fatigue among individuals with high daily screen exposure. Controlled trials with larger and more diverse populations are recommended to establish causal effectiveness.

## INTRODUCTION

The rapid digitalization of academic and professional environments has substantially increased daily exposure to screen-based technologies, particularly among university students and staff who rely on digital platforms for coursework, communication, and administrative tasks. Recent evidence suggests that prolonged screen exposure is strongly linked to musculoskeletal discomfort, alterations in postural alignment, and decreased overall well-being^1-3^. Repetitive upper-extremity activity, static seated postures, and suboptimal workstation habits contribute to the growing prevalence of musculoskeletal symptoms in digitally engaged populations.

Beyond physical consequences, excessive screen exposure is closely linked to oculomotor fatigue, visual discomfort, attentional instability, and digital eye strain (DES), a syndrome whose prevalence has increased sharply over the past decade^4-7^. Digital media use has also been shown to impair sleep patterns and elevate psychological stress, underscoring the multidimensional health impact of intensive screen engagement^5,8^. These emerging findings highlight the complex interplay between visual, cognitive, and musculoskeletal demands in digital environments.

Increasing evidence suggests that extended screen use disrupts the integration of multisensory systems, particularly visual, vestibular, and proprioceptive inputs that are essential for maintaining postural control and sensorimotor ­regulation^2,9,10^. Altered cervical proprioception and diminished head-eye coordination, frequently observed in screen-exposed individuals, may impair reflexive pathways such as the cervico-ocular and cervicocollic reflexes, contributing to balance instability and sensorimotor inefficiency. Despite these risks, multimodal interventions addressing the multisensory consequences of prolonged screen exposure remain limited.

While prior studies have examined isolated approaches such as general stretching, visual exercises, or physical activity programs, few have investigated virtual or hybrid interventions that simultaneously target sensorimotor, oculomotor, and postural domains^3,11^. Given the rapid evolution of remote learning, hybrid work, and digital communication platforms, there is a growing need for accessible, evidence-based strategies that address the interconnected nature of postural awareness, proprioceptive acuity, and oculomotor function^7,11,12^.

However, the literature remains limited regarding multimodal virtual interventions that integrate sensorimotor, oculomotor, and postural training within real-time supervised digital platforms. Most existing studies have focused on single-component exercise approaches rather than comprehensive and integrated protocols, which limits their applicability to the multidimensional demands of prolonged screen-based activity^7,11,12^.

Therefore, the present study aimed to evaluate the effects of a virtually delivered multimodal exercise intervention, including sensorimotor, oculomotor, and postural components, on postural awareness, musculoskeletal discomfort, visual fatigue, and anxiety among university students and staff with high daily screen exposure. By addressing a multifaceted health concern with an integrated approach, this study aims to contribute updated and clinically relevant evidence to support preventive strategies that mitigate the physical and psychological consequences of digital technology use.

## METHODS

### Study design and setting

This cross-sectional descriptive study included young adults (students and staff) from Alanya Alaaddin Keykubat University, selected through convenience sampling based on voluntary participation. The sample's homogeneity was intentional, reflecting the practical scope of a TÜBİTAK 2209 student research project. Accordingly, demographic diversity was limited in terms of age, occupation, and education level. This controlled homogeneity aimed to enhance internal validity by minimizing the influence of extraneous variables such as occupational differences, ergonomic diversity, and age-related variability in sensorimotor function. Nevertheless, we recognize that such intentional homogeneity restricts generalizability to wider screen-exposed adult populations.

### Eligibility criteria

The inclusion criteria included being aged between 18 and 65 years, having a minimum of 6 h per day of cumulative screen exposure, including computer, smartphone, and tablet use for academic, professional, or recreational purposes.

Exclusion criteria were a history of spinal surgery or trauma, neurological deficits, vestibular disorders, any cardiopulmonary or musculoskeletal conditions affecting physical performance, and pregnancy.

Recruitment: Participants completed an 8-week program that included twice-weekly live sessions (20–25 min each) via WhatsApp Web and a 15-min pre-recorded video comprising the exercises listed in Table 1. The video was shared with participants via WhatsApp for daily self-practice. Exercises were instructed and demonstrated by a certified physiotherapist specializing in musculoskeletal rehabilitation. Participants were encouraged to repeat the video session at least three times a day and to record their adherence through weekly feedback forms. The overall attendance rate for live sessions was approximately 88%. Based on weekly adherence logs, participants completed a mean of 2.4±0.8 daily video repetitions and attended 86% of all scheduled live sessions, yielding an overall adherence rate of 82% across the 8-week program (Table 1).

**Table 1:** Exercise protocols and delivery parameters.

Step	Description	Type
Group 1: Oculomotor sensorimotor exercises		
1	Head in neutral, fixate on colored object in right hand and count to 10, then switch to object in left hand and count to 10.	Fixation exercise
2	Head in neutral, follow colored object in right hand as it moves from right to left and back without moving head.	Smooth pursuit
3	With colored object held to the left, fixate with eyes while rotating the head right and left.	Adaptation X1
4	Fixate on colored object and rotate arm and head in opposite directions.	Adaptation X2
Group 2: Postural exercises		
1	Wrist and finger stretch with palms outward, arms overhead while keeping elbows extended. Hold for 5 s, repeat three times.	Stretching
2	Shoulder girdle stretch by pulling one arm medially behind the head using the opposite hand. Include lateral trunk flexion. Repeat bilaterally three times.	Stretching
3	With hands on lower back, press lumbar spine forward while extending neck. Then cross legs, rotate trunk and head toward upper leg. Repeat three times bilaterally.	Stretching
4	Move both arms backward: one at 135° flexion (palm forward) and the other at 45° flexion (back of hand forward). Hold for 5 s. Repeat bilaterally three times.	Stretching
5	Elevate shoulders as high as possible and then slowly depress while keeping elbows extended. Repeat three times.	Scapular Elevation/Depression
6	With arms at 90° abduction and elbows extended, bring arms slowly together to 90° flexion and return. Repeat three times.	Scapular abduction/adduction
7	Unilateral trapezius stretch by stabilizing upper trapezius with opposite hand and laterally flexing the head. Hold for 5 s, repeat three times bilaterally.	Trapezius stretch
8	Diaphragmatic breathing: Place left hand on chest and right hand on upper abdomen. Breathe in through the nose, inflating abdomen without chest movement. Repeat five times.	Breathing exercise

Participants completed an 8-week program including twice-weekly live sessions (20–25 min each) via WhatsApp Web and a 15-min pre-recorded video composed of the exercises listed in Table 1. The video was shared with participants daily for self-practice. All sessions were instructed and demonstrated by a certified physiotherapist (MSc) specializing in musculoskeletal rehabilitation. Participants were encouraged to repeat the video session at least three times per day and to record their adherence through weekly feedback forms. Based on adherence logs, participants completed a mean of 2.4±0.8 daily video repetitions and attended 86% of all scheduled live sessions, yielding an overall adherence rate of 82% across the 8-week program.

The data collection stage was conducted using a structured evaluation form administered within an internet environment. The evaluation form was sent via a Google Forms link over various communication routes (Messaging, WhatsApp, etc.) and social media platforms (Facebook, Instagram, etc.).

### Assessment and data collection

Demographic Information and Eye Fatigue Assessment: Data were collected through a structured, three-part online questionnaire distributed via Google Forms. The first section captured demographic data (e.g., age, gender) and visual environment factors such as daily screen time, device type, and use of glasses or screen filters.

The second section assessed eye-related symptoms (e.g., burning, dryness, blurred vision, headache) experienced after 1 h of screen exposure, rated on a five-point frequency scale from "never" to "often." The final section evaluated symptom changes during the online education period, with responses ranging from "decreased a lot" to "increased a lot"^13^.

### Postural Habits and Awareness Scale

The PHAS, developed by Bayar et al. and validated in Turkish, evaluates individuals’ postural habits and awareness in daily activities. The scale consists of 19 items rated on a five-point Likert scale ("1=strongly disagree" to "5=strongly agree"), with seven reverse-scored items. It comprises two subscales: postural habits (max score=35) and postural awareness (max score=60), totaling a maximum score of 95. Higher scores reflect better posture and greater awareness in activities such as standing, sitting, lying, shopping, and lifting^14^.

Cornell Musculoskeletal Disorders Questionnaire (CMDQ): The CMDQ, validated in Turkish by Erdinc et al., was used to assess musculoskeletal symptoms over the past week^15^. It evaluates the frequency, intensity, and work-related impact of pain or discomfort in specific body regions. Participants rated how often they experienced symptoms, their severity, and whether these affected their work. Individual risk scores were calculated based on their responses.

Rapid Office Strain Assessment (ROSA): The ROSA tool was used to evaluate participants’ ergonomic risk levels related to office workstation design. This observational instrument assesses postural risks associated with chairs, desks, screens, keyboards, mice, and telephones, based on established methods such as Rapid Upper Limb Assesment (RULA) and Rapid Entire Body Assesment (REBA)^16^.

In this study, ROSA was included as a secondary outcome measure to monitor potential variations in ergonomic risk during the intervention. Because the exercise program did not involve any physical modification of workstation components, a substantial change in ROSA scores was not expected.

Each participant's workstation was assessed on-site by a trained observer using the standardized ROSA diagrams and matrix. Risk scores were calculated across four domains:

- Chair: Adjustability and lumbar support features
- Screen and phone: Alignment, lighting, and usage duration
- Keyboard and mouse: Posture and frequency of use
- Overall risk: Combined total of all components

Higher ROSA scores indicate elevated ergonomic risk. Following the reviewer's recommendation, the manuscript now explicitly presents the risk thresholds defined by Sonne et al.^16^:

- ≥7: High ergonomic risk
- 5–7: Moderate risk
- 0–4: Low risk

### Spielberger State and Trait Anxiety Inventory

The STAI was used to assess participants’ anxiety levels, consisting of two subscales: STAI-I (state anxiety) and STAI-II (trait anxiety), each with 20 items. Both forms include direct and reverse-coded items, and scoring is based on a standardized formula. Higher total scores reflect greater anxiety. The Turkish adaptation of the STAI was validated between 1974 and 1977^17^ and it remains a widely accepted tool for evaluating emotional state variability in psychological research.

Statistical analysis: Statistical analysis was performed using the Statistical Package for the Social Sciences (SPSS), version 25.0 (IBM Corp., Armonk, NY, USA). The normality of the data distribution was evaluated using the Shapiro-Wilk test. Paired samples t-test was used for normally distributed variables, and a Wilcoxon signed-rank test was used for non-normally distributed variables. Demographic findings, clinical findings, and delta (Δ) values were reported using descriptive statistics, including mean±standard deviation for normal distribution data and median (interquartile range) for the data that has not been normally distributed. The delta (Δ) values are calculated from the differences between the pre-test and post-test. A significance level of p<0.05 was accepted in all analyses.

Ethics statement: Ethical approval was obtained from the Alanya Alaaddin Keykubat University Non-Interventional Clinical Research Ethics Committee (09/07/2024, Decision No: 2024/05), and the study was conducted in accordance with the Declaration of Helsinki. The study was supported by the Scientific and Technological Research Council of Turkey (TÜBİTAK 2209-A, Project No: 1919B012324064).

Participants were informed about the study through a WhatsApp group meeting, after which they received consent forms via an online platform. Both verbal and written consents were obtained prior to the commencement of data collection.

## RESULTS

A total of 57 volunteer participants were included in the study. The study comprised 70.2% female participants (n=40) and 29.8% male participants (n=17). The mean age of the participants was 23.35±1.2 years, with an age range of 22–27 years. The mean height was 168.63±8.22 cm, body weight was 64.84±13.14 kg, and body mass index (BMI) was 22.65±3.27 (Table 2).

**Table 2:** Demographic information.

		n	%
Gender	Woman	40	70.2
	Man	17	29.8
Age	Mean±SD	23.35±1.2	
	Med (IQR)	23 (22.5–24)	
	Min–max	22–27	
Weight	Mean±SD	64.84±13.14	
	Med (IQR)	62 (55.5–72.5)	
	Min–max	42–97	
Height	Mean±SD	168.63±8.22	
	Med (IQR)	166 (163–174.5)	
	Min–max	149–188	
BMI	Mean±SD	22.65±3.27	
	Med (IQR)	22.66 (19.55–25.54)	
	Min–max	16.8–29.74	
Level of education	University	57	100.0
Have you been using a computer at home in the last 6 months?	No	15	26.3
	Yes, less than 1 h a day	27	47.4
	2 h or more than 2 h	15	26.3
Computer use duration of more than 1 h per day (in years)	Mean±SD	3.88±2.78	
	Med (IQR)	4 (3–5)	
	Min–max	0–20	
How many days a week have you been working in the past 6 months?	Mean±SD	4.51±2.34	
	Med (IQR)	4 (4–5)	
	Min–max	2–20	
Do you take breaks while using the computer?	No	20	35.1
	Yes	37	64.9
Do you exercise?	I do it regularly several times a week	15	26.3
	Occasionally	34	59.6
	No	8	14.0
Do you smoke	I have never smoked	23	40.4
	I used to smoke, but I quit	10	17.5
	I currently smoke	24	42.1
Do you have any chronic illnesses?	Yes	57	100.0
Which chronic illness do you have?	Headache	26	45.6
	Hypertension	4	7.0
	Eczema	4	7.0
	Diabetes	1	1.8
	Eye diseases	15	26.3
	Thyroid disorders	1	1.8
	Cervical disc herniation	5	8.8
	Lumbar disc herniation	1	1.8

n: frequency; %: percent; SD: standard deviation; BMI: body mass index; Med (IQR): median (25th–75th percentiles); min–max: minimum–maximum values.

All participants were university students, and their education level was undergraduate (100%). Regarding their computer use over the last 6 months, 26.3% stated that they had never used a computer, 47.4% reported using a computer for less than 1 h a day, and 26.3% stated that they used a computer for 2 h or more a day. The average duration of computer use was reported as 3.88±2.78 years. The average number of working days per week was 3.95±2.86 days. Sixty-four point nine percent of the participants stated that they took breaks during computer use (Table 2).

In terms of exercise habits, 26.3% exercised regularly ­several times a week, 59.6% exercised occasionally, and 14.0% did not exercise at all. In terms of smoking habits, 40.4% had never smoked, 17.5% had smoked before but quit, and 42.1% were current smokers (Table 2).

All participants (100%) had at least one chronic disease. The most frequently reported chronic diseases were headache (45.6%), eye diseases (26.3%), cervical hernia (8.8%), hypertension (7.0%), eczema (7.0%), diabetes (1.8%), thyroid ­diseases (1.8%), and lumbar hernia (1.8%) (Table 2).

No significant change was observed in any of the ­dimensions of the ROSA scale (p>0.05) (Table 3).

**Table 3:** Within-group differences in pre- and post-test scores.

	Pre-test	Post-test	Intra group p	Delta Δ
Rosa A	5 (4–5)	5 (4–5)	0.414 (z=-0.816)	0 (0–0)
Rosa monitor B	1 (1–1)	1 (1–1)	1 (z=0)	0 (0–0)
Rosa C	4 (4–4)	4 (4–4)	0.48 (z=-0.707)	0 (0–0)
Rosa toplam	10 (9–10)	10 (9–10)	0.285 (z=-1.069)	0 (0–0)
PHAS FACTOR 1	24.74±3.9	25.77±3.83	**0.012** * **(t=-2.585)**	-1 (-4–1)
PHAS FACTOR 2	15 (14–16)	16 (12–16)	0.682 (z=-0.41)	0 (-2–2)
PHAS FACTOR 3	12 (11–14)	14 (12–15)	**0.0001** * **(z=-4.148)**	-2 (-3–0)
PHAS FACTOR 4	8.28±1.92	7.89±2.01	0.199 (t=1.3)	0 (-1–2)
CMDQ Part 1	43 (37.5–52)	34 (25–43.5)	**0.0001** * **(z=-6.576**)	9 (7.5–12)
CMDQ Part 2	46 (41–52.5)	40 (32.5–45.5)	**0.0001** * **(z=-6.256)**	6 (2.5–8)
CMDQ Part 3	33.49±8.22	27.51±8.38	**0.0001** * **(t=14.865)**	6 (4–8)
STAI-I State Anxiety	47 (45–49)	48 (46–50)	**0.005** * **(z=-2.804)**	-1 (-2–0)
STAI-II Trait Anxiety	47 (46–49)	47 (45–48.5)	0.301 (z=-1.034)	1 (-1–2)
ASTHENOPIA	1 (0–2)	0 (0–1.5)	**0.001** * **(z=-4.185)**	0 (0–1)

* p<0.05 indicates a statistically significant change and is represented in bold; SD: standard deviation; Med (IQR): median (25th–75th percentiles); z: Wilcoxon Signed-Rank Test. Normally distributed variables were analyzed with paired-samples t-test and presented as mean±SD. Non-normally distributed variables were analyzed with Wilcoxon signed-rank test and presented as median (IQR). Delta (Δ) values were reported accordingly. PHAS: Postural Habits and Awareness Scale; CMDQ: Cornell Musculoskeletal Discomfort Questionnaire; STAI-I: State-Trait Anxiety Inventory-I; STAI-II: State-Trait Anxiety Inventory-II.

A significant increase was observed in the sub-dimensions of Posture Habit and Awareness (Factor 1) and Positional Awareness (Factor 3) (p<0.05), indicating that participants experienced positive improvements in both posture awareness and postural position awareness in their daily lives. No significant change was found in the Awareness of Factors Disturbing Posture (Factor 2) and Ergonomic Awareness (Factor 4) sub-dimensions (p>0.05) (Table 3).

A significant decrease was observed in all sections of the CMDQ (p<0.05), indicating a decrease in musculoskeletal complaints among the participants (Table 3).

There was a significant increase in the Spielberger STAI (p<0.05), whereas no significant change was observed in the trait anxiety level (p>0.05) (Table 3).

Eye fatigue showed a statistically significant reduction following the intervention (pre: 1.79±2.41; post: 1.19±2.13), confirmed by the Wilcoxon test (z=-4.185, p<0.001). Median scores decreased from 1.00 (0.00–2.00) to 0.00 (0.00–1.50) (Table 3).

These values correspond exactly to those reported in Table 3.

## DISCUSSION

This study demonstrated that a virtually delivered multimodal exercise program, combining sensorimotor, oculomotor, and postural components, produced significant improvements in postural awareness, musculoskeletal complaints, and visual fatigue among university students and staff who were exposed to prolonged daily screen use. The marked reductions in Posture Habit and Awareness (Factor 1), Positional Awareness (Factor 3), and CMDQ scores are consistent with recent findings that short, structured multimodal exercise programs can effectively enhance proprioceptive efficiency, correct postural habits, and reduce musculoskeletal symptom burden in digitally engaged populations^2,3^. Following the widespread adoption of virtual learning and hybrid work models, simple online feedback-based exercise routines have also been shown to improve alignment of the head, shoulders, and trunk^1-3,11^ These findings collectively highlight the potential of virtual sensorimotor-based interventions as accessible strategies for addressing the biomechanical consequences of prolonged screen exposure.

The significant reduction in Asthenopia scores (z=-4.185, p<0.001) provides robust evidence that the oculomotor and sensorimotor components of the virtual program effectively alleviated visual fatigue. This finding aligns with recent studies demonstrating that targeted oculomotor training reduces DES by improving accommodative efficiency, blink regularity, and ciliary muscle control^4-7^. Similar improvements in visuocognitive and oculomotor task performance following virtual reality–based training have also been demonstrated in both peri- and post-menopausal adults, highlighting the neuroplastic potential of visually guided digital interventions^18^. Given the growing prevalence of screen-related visual symptoms among young adults, the improvement observed in our study reinforces the value of accessible, virtual eye–neck coordination exercises in mitigating visual discomfort^1,2^.

The absence of significant changes in ROSA scores should be interpreted within the context of both the study population and the inherent characteristics of the ROSA assessment tool. Most participants were young adults whose daily routines did not primarily involve sustained desk-based work. Consequently, their cumulative ergonomic exposure was limited, reducing the likelihood of measurable changes over the short intervention period. ROSA was originally designed to evaluate static workstation configurations and sustained postural demands typical of full-time office workers^6,19^. In this population, baseline ROSA values (mean≈9.5) reflected suboptimal workstation setups rather than accumulated ergonomic strain.

Moreover, ROSA primarily quantifies equipment-related and layout-based ergonomic parameters, with limited sensitivity to behavioral or perceptual adaptations, which are the specific targets of virtual sensorimotor–postural training. Thus, improvements in postural awareness identified through PHAS may not translate into short-term changes on a tool focused on static workstation features. Future studies may benefit from combining ROSA with subjective ergonomic perception scales or digital posture-tracking systems to capture subtle changes in ergonomic behavior among individuals with low-intensity workstation exposure.

The unexpected increase in state anxiety (STAI-I) warrants careful consideration. Several mechanisms, supported by emerging evidence, may explain this response. Virtual and hybrid learning environments are known to increase cognitive load, attentional demand, and self-focused monitoring, contributing to short-term elevations in anxiety^8,12^. Virtual simulator-based studies have further demonstrated that visual distraction and increased attentional load significantly influence motor reaction time and postural responses, indicating that visually demanding digital tasks can induce heightened neurocognitive strain^20^. Live group-based virtual sessions may amplify performance-related self-awareness and social evaluative concerns, particularly when participants feel observed by peers^21^.

Additionally, early engagement in body-focused, awareness-based, and sensorimotor practices can temporarily heighten interoceptive awareness, a mechanism shown to intensify anxiety during the initial stages of posture- and awareness-based interventions^7,9,10^. This response may be further amplified in visually and cognitively engaging digital environments. A recent systematic review of virtual reality-based interventions during midlife reported consistent enhancements in visuomotor coordination and cognitive engagement, highlighting the increased attentional and interoceptive demands imposed by immersive digital exercise platforms^22^. For young adults, who may be less familiar with interoceptive training, increased internal attention can magnify uncertainty regarding task performance and amplify transient anxiety responses.

Digital fatigue may also contribute. Individuals with high baseline screen exposure may experience an added mental load during virtual sessions, consistent with meta-analytic evidence linking videoconferencing to "Zoom fatigue" and elevated physiological^10^ arousal^8,12^. Importantly, anxiety scores remained below clinically significant thresholds and did not affect adherence, indicating a transient and manageable response rather than an adverse psychological outcome.

Future interventions may reduce anxiety reactivity by incorporating structured onboarding, adaptive difficulty progression, real-time guidance, and more opportunities for clarifying expectations and performance criteria. Tracking engagement levels and integrating qualitative feedback may further enhance psychological safety.

Collectively, the results demonstrate that virtual multimodal exercise programs represent a promising, accessible, and multidimensional approach to addressing the physical and visual demands of digital environments. By simultaneously targeting sensorimotor, oculomotor, and postural pathways, these interventions may serve as effective tools for mitigating screen-related musculoskeletal, postural, and visual complaints.

However, the absence of a control group and the short duration of the intervention limit the ability to draw causal inferences. Future studies should employ randomized controlled designs, blinded assessments, longer follow-up periods, and integrated measures of engagement to more rigorously evaluate effectiveness and understand the psychological impact of hybrid virtual exercise programs.

### LIMITATIONS

This study has several limitations that should be taken into account when interpreting the findings. First, the single-group pre–post design and absence of blinding limit causal inference. These constraints were largely due to the feasibility requirements of the TÜBİTAK 2209 student research framework, which provides restricted time and resources for implementing parallel control groups. Within this context, the study was intentionally structured as a preliminary feasibility investigation to explore the applicability, safety, and potential direction of intervention effects. Nevertheless, future research should incorporate randomized controlled designs with blinded assessors and larger samples to strengthen internal validity.

Second, the interpretation of ROSA scores warrants caution. The intervention focused on sensorimotor, oculomotor, and postural awareness, rather than workstation ergonomics, and no physical adjustments were made to chairs, screens, or desks. As such, stability in ROSA scores was expected and aligns with previous studies, which indicate that ROSA primarily captures static, equipment-related ergonomic parameters rather than behavioral or perceptual changes^6^. Additionally, the young, non-desk-based nature of the sample limits the relevance of workstation-based ergonomic measures, further reducing the likelihood of detecting short-term ergonomic changes.

Third, the homogeneity of the sample limits ­generalizability. Participants were predominantly young university students with similar academic routines and relatively low cumulative ergonomic load. While this homogeneity helped reduce confounding factors and improve internal consistency for a pilot study, it limits extrapolation to older adults or professional desk workers. Future investigations should include more diverse populations with varying ergonomic exposures.

Fourth, adherence and engagement were self-reported, which introduces potential bias. Although participation was ­encouraged through supervised sessions and daily video ­guidance, objective measurement tools such as engagement trackers or wearable sensors were not utilized. Reliance on self-reported symptoms, including headaches or eye discomfort, may also have introduced minor confounding effects.

Finally, the relatively short duration and moderate intensity of the program may not have been sufficient to elicit sustained psychological or neurophysiological adaptations, particularly regarding anxiety outcomes. Extending intervention length, refining task difficulty progression, and integrating real-time feedback may enhance both psychological and physical outcomes in future programs.

## Data Availability

The datasets generated and/or analyzed during the current study are available from the corresponding author upon reasonable request, subject to ethical approval and institutional regulations.
